# A new species of Andean semiaquatic lizard of the genus *Potamites* (Sauria, Gymnophtalmidae) from southern Peru

**DOI:** 10.3897/zookeys.168.2048

**Published:** 2012-01-27

**Authors:** Germán Chávez, Diego Vásquez

**Affiliations:** 1 Centro de Ornitología y Biodiversidad (CORBIDI). Calle Santa Rita 135, Urb. Los Huertos de San Antonio, Lima 33, Peru

**Keywords:** *Potamites*, Cusco, Ayacucho, Peru

## Abstract

We describe a new lizard species of the genus *Potamites* from the montane forests of the Cordillera de Vilcabamba (Cusco region) and Apurimac River valley (Ayacucho region), between 1500 and 2000 meters of elevation, in southern Peru. The new species is distinguishable from all other species of the genus mainly by having highly keeled scattered scales on dorsum and females lacking femoral pores.

## Abstract (sp)

Describimos una nueva especie de lagartija del genero *Potamites* de los bosques montanos de la Cordillera de Vilcabamba y del Valle del río Apurímac, entre los 1500 y 2000 metros de elevación, en las regiones de Cusco y Ayacucho respectivamente, del sur de Peru. La nueva especie es distinguible de todas las otras especies del genero principalmente por tener escamas altamente quilladas y desordenadas en el dorso y por no presentar poros femorales en las hembras.

## Introduction

The semi aquatic lizards of the genus *Potamites* were considered as *Neusticurus* for many years, this genus included eleven species, however *Neusticurus* is currently represented only by five species and the other six are included in the genus *Potamites* (Doan and Castoe 2005). Two species of *Potamites* occur formerly in Peru: *Potamites ecpleopus* and *Potamites strangulatus*; *Potamites strangulatus* has two recognized subspecies: *Potamites strangulatus strangulatus* and *Potamites strangulatus trachodus*, recorded in the Amazonian lowlands between 100 and 800 m elevations in the centre and the north respectively (Uzell 1966) and easily distinguishable by the absence or presence of tubercles on the flanks. Additionally another species of the genus, *Potamites juruazenzis*, was described from rio Juruá, Acre state, at southwestern Brazil (Avila-Pires and Vitt 1998), but even though it has never been recorded in Peru, its occurrence there is expected due to the proximity of the type locality to the borderline with the Ucayali Region. *Potamites ocellatus* (Sinitsin 1930) was described with a holotype from Rurrenabaque (Beni region, Bolivia) and 54 paratypes from Chanchamayo and Perené valleys (Junin, Peru). However, the variation in the paratypes was not included in the description, and given the distance between both sites (approximately 1000 km airline), they were questioned to belong to the same species. Sinitsin in a footnote explains that the new form would be analized by Charles E. Burt in another paper. In the new publication, Burt and Burt (1931) conclude that *Potamites ocellatus* is a subspecies of *Potamites ecpleopus* because the scutellation and measurements are similar to *Potamites ecpleopus*. Some years later, Uzell (1966) proposed the subspecies *Potamites ecpleopus ocellatus* to be a synonym of *Potamites ecpleopus* and later on Vanzolini (1995) resurrected *Potamites ocellatus* and elevated it to the species level (but in reference to the Bolivian holotype only). Even though, the genus *Potamites* still presents several taxonomic uncertainties, it also proves that its diversity is also underestimated and poorly studied. Recent surveys carried out during 2010 gave as a result the discovery of a new species of *Potamites* from southern Peru, which is described herein.

## Material and methods

The description format for the new species generally follows that of Uzell (1966), Vanzolini (1995) and Avila-Pires and Vitt (1998). For the comparisons, we used the descriptions from all *Neusticurus* and *Potamites* species known in the literature: data for most *Neusticurus* and *Potamites* were taken from Uzzell (1966), data for *Potamites juruazensis* was taken from Avila-Pires and Vitt (1998), for *Potamites ocellatus* from Vanzolini (1995), data of *Neusticurus tatei* from Barrio-Amorós and Brewer-Carias (2008) (for a detailed list of specimens reviewed see Appendix 1). Nomenclature of scale characters follows that of Uzell (1966) and Köhler and Lehr (2004). Scale sizes were measured using precision calipers and were rounded to the nearest 0.1 mm. For characters recorded on both sides, the condition on the right side is presented first. Everted hemipenes were fixed with formalin 3.7%. The abbreviation for the museum collection is CORBIDI (Centro de Ornitología y Biodiversidad) and GPS coordinates were taken using the geodetic datum WGS84.

## Results

### Potamites montanicola sp. n.

urn:lsid:zoobank.org:act:1B259770-6CF5-4236-9E62-785266E6ECFC

http://species-id.net/wiki/Potamites_montanicola

**Figure 1.**
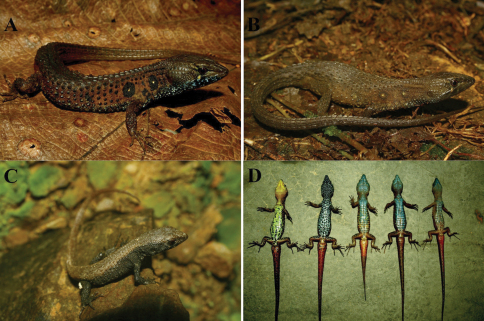
*Potamites montanicola*, new species from southern Peru. Holotype male (CORBIDI 08322) **A** female (CORBIDI 08328) **B** uncollected juvenile **C** ventral view of males of the type series, from left to right: CORBIDI 08324, CORBIDI 08322 (holotype), CORBIDI 08325, CORBIDI 08326, CORBIDI 08335 **D**

Figures 1 [Fig F2] [Fig F3] –4

#### Holotype.

**Figure 2. F2:**
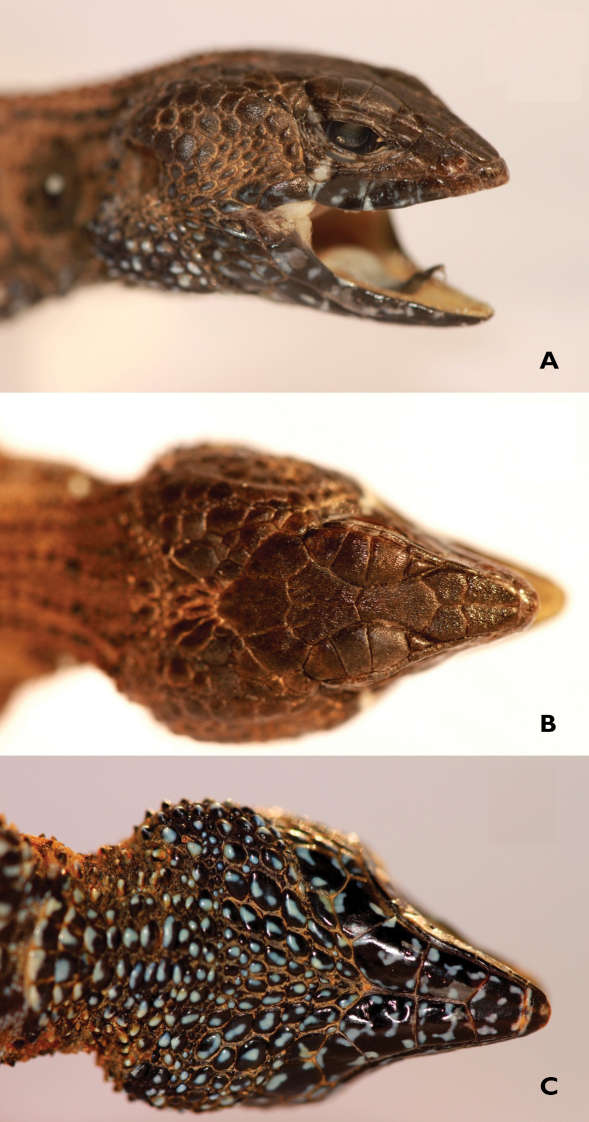
Lateral **A** dorsal **B** and ventral C views of the head of *Potamites montanicola* holotype (CORBIDI 08322)

(Fig. 1a; 2a–b; 3a,d) Adult male (CORBIDI 08322), Peru, Cusco Region, La Convención Province, 4.8 km E of Alto Shimá Native Community (12°34'16.4'S, 73°09'42.3'W), 1577 m elevation, collected by Germán Chávez and Diego Vasquez on 3 December 2010.

#### Paratypes.

**Figure 3. F3:**
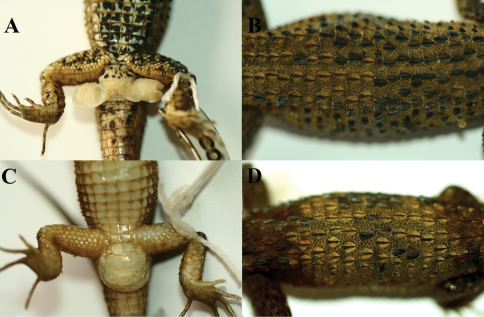
Distinguishable characters of *Potamites montanicola.* Femoral pores and dorsal scutellation in the holotype (CORBIDI 08322) **A–B** absence of femoral pores and dorsal scutellation in female (CORBIDI 08328) **C–D**

(Fig. 1b, d; 3b, c; 4a) CORBIDI 08324-27, 08335 (all adult males), 08328, 08334, 08336, (all adult females), same data as holotype, CORBIDI 06957 (adult male), Peru, Ayacucho Region, La Mar Province, Cajadela Community (12°57'27.8'S, 73°36'22.4'W), 2098 m elevation, collected by Karla García on 23 August 2010.

**Figure 4.**
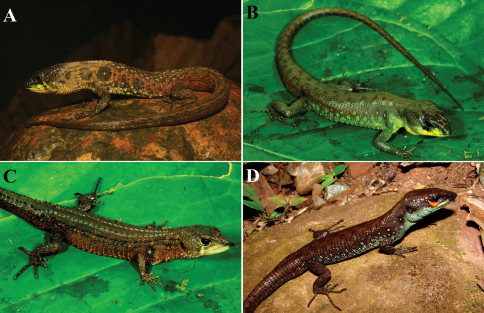
*Potamites* species from Peru. *Potamites montanicola* (CORBIDI, 08324) **A** *Potamites strangulatus strangulatus* from Cordillera de Kampankis, Amazonas*,* northern Peru (not collected individual) **B** photo by Alessandro Catenazzi; *Potamites ecpleopus* fromCordillera de Kampankis, Amazonas, northern Peru (CORBIDI 09516) **C** photo by Alessandro Catenazzi; *Potamites strangulatus trachodus* from Cordillera Escalera, San Martin, northern Peru (CORBIDI 06368) **D** photo by Pablo J. Venegas.

#### Diagnosis.

Assigned to *Potamites* sensu stricto by having a tongue with imbricate scale-like papillae, movable eyelids, external ear and heterogeneous dorsal scalation. 1) Head acuminate from dorsal and lateral views, frontonasal length usually equal or slightly shorter than frontal length; (2) nasoloreal suture present; (3) supraoculars four, anteriormost supraocular not fused with anteriormost superciliary, all supraoculars separated from superciliaries; (4) superciliary series complete, usually four; (5) supralabial-subocular fusion absent; (6) postoculars three; (7) postparietals three; (8) genials in two pairs, transverse sutures perpendicular with respect to midline of body; (9) dorsal scales rectangular, juxtaposed, granular and keeled; (10) transverse dorsal count (enlarged rows at midbody) at midbody 36–42 in both sexes; (11) longitudinal dorsal keeled scales count 32–37 in both sexes; (12) longitudinal ventral count 21 – 23 in both sexes; (13) femoral pores in males 19–21, absent in females; two scales between femoral pores; (14) subdigital scales on 4th finger 13–17, on 4th toe 22–27; (15) forelimb reaching anteriorly to fourth supralabial; (16) Tail slightly compressed with two rows of lateral scales per two ventral caudal scales; (17) hemipenis acapitate; flounces lacking calcified spines and forming two chevrons on distal half of hemipenis while basal half is covered with 5 transverse flounces; some asulcate flounces separated by a small expansion pleat; sulcate flounces about as wide as asulcate flounces; sulcus spermaticus single, flanked by a broad naked expansion pleat widened distally; (18) dorsum dark brown; lateral ocelli present in two or three pairs in males, usually absent in females; ventral color pattern pale blue or yellow with black blotches in males and creamy white in females; (19) transparent lower palpebral disc an undivided oval; (20) prefrontals present.

*Potamites montanicola* is easily distinguished of all other *Potamites* and *Neusticurus* species by having highly keeled scales scattered all over the dorsum (all species have either tubercles or keeled scales forming longitudinal rows from neck to the insertion of the hind limbs, or lack of them) and by females lacking femoral pores (only some female specimens of the type series in *Potamites juruazensis* lack femoral pores). Of all *Potamites* species, *Potamites montanicola* best resembles *Potamites ecpleopus*, *Potamites juruazenzis* and *Potamites ocellatus*. It differs from *Potamites ecpleopus* by having a lower number of keeled scales on dorsum (see specimens reviewed in Appendix 1): 32–37 (vs 36–45), a higher number of scales around midbody: 43–50 (vs 34–46), frenocular scale pentagonal (vs triangular) and a lower number of femoral pores bearing 19–21 in males and lacking in females (vs 25–48 in males and 1–15 in females). Differs from *Potamites juruazenzis* by having a higher number of scales around midbody: 42–50 (vs 31–40), bearing scattered dorsal highly keeled scales (vs bearing four longitudinal rows of dorsal tubercles), a higher number of lamellae of fourth toe: 22–27 (vs 16–22), a higher number of femoral pores in males: 19–21 (vs 10 -16) and by lacking femoral pores in females (vs 0–2 femoral pores). Differs from *Potamites ocellatus* by its smaller size: 68.6 mm as maximum SVL in males (vs 75 mm in *Potamites ocellatus*), dorsal highly keeled scales present (vs flat dorsal tubercles present), temporal region covered by medium size polygonal scales (vs covered by large scales interspersed with granules) and has a lower number of femoral pores in males: 19–21 (vs 41).

Furthermore, *Potamites montanicola* differs from other *Potamites* and *Neusticurus* species in its smaller size, having a maximum SVL of 68.6 mm in males (vs 117 mm in *Neusticurus bicarinatus*, 121 mm in *Neusticurus medemi*, 104 mm in *Neusticurus racenisi*, 94 mm in *Neusticurus rudis*, 87 mm in *Potamites strangulatus* and 94 mm in *Neusticurus tatei*) and 56.1 mm in females (vs 96 mm in *Neusticurus bicarinatus*, 79 mm in *Potamites cochranae*, 107 mm in *Neusticurus medemi*, 94 mm in *Neusticurus racenisi*, 89 mm in *Neusticurus rudis*, 76 mm in *Potamites strangulatus* and 85 mm in *Neusticurus tatei*), bearing dorsal crests (absent in *Neusticurus racenisi*, *Neusticurus rudis* and *Potamites strangulatus*), bearing tubercles on flanks (absent in *Potamites cochranae*, *Neusticurus medemi*, *Neusticurus racenisi*, *Potamites strangulatus* and *Neusticurus tatei*), having a superficial tympanum (deep in *Neusticurus bicarinatus*, *Neusticurus medemi*, *Neusticurus racenisi* and shallow in *Potamites cochranae* and *Neusticurus rudis*), having a low number of femoral pores in males:19–21 (vs 26–30 in *Potamites apodemus*, 40–62 in *Neusticurus bicarinatus*, 58–64 in *Neusticurus medemi*, 62–72 in *Neusticurus racenisi*, 32–46 in *Neusticurus rudis*, 45–59 in *Potamites strangulatus* and 60–61 in *Neusticurus tatei*) and lacking femoral pores in females (femoral pores present in all *Potamites* and *Neusticurus* excepting some individuals of *Potamites juruazensis*).

#### Description of the holotype.

Adult male (CORBIDI 08322), body long, laterally compressed, SVL 68.64 mm; tail (complete) length 107.01 mm, axilla to groin distance 32.90 mm; head length 16.40 mm; head width 10.29 mm; shank length 10.27 mm. Head scales smooth; rostral scale wider (2.57 mm) than long (1.28 mm), higher than adjacent supralabials, in contact with frontonasal, nasoloreal, and first supralabials posteriorly; frontonasal almost squarish, slightly longer (2.57 mm) than wider (2.27 mm), widest posteriorly, in contact with nasoloreal and frenocular laterally, prefrontals posteriorly; nasoloreal almost triangular, apex in contact with rostral, nasoloreal suture present; prefrontals present, in contact with each other medially, in contact with anteriormost superciliary and anteriormost supraocular, frontal posteriorly; frontal longer (3.79 mm) than wider (2.31 mm), anterior suture angular with point directed anteriorly, lateral sutures straight, posterior suture angular with point slightly directed posteriorly, in contact with first and second supraocular laterally, frontoparietals posteriorly; frontoparietals pentagonal, in contact with third and fourth supraocular, parietals and interparietal posteriorly; supraoculars four, none in contact with ciliaries; superciliary series complete, generally four, anteriormost superciliary not fused with anteriormost supraocular; interparietal pentagonal, longer (3.63 mm) than wider (2.80 mm), in contact with parietals laterally, postparietals posteriorly; parietals pentagonal, in contact with fourth supraocular anterolaterally, temporal scales laterally, dorsalmost postocular, postparietals posteriorly; postparietals ten, polygonal, boardering parietals and interparietal; palpebral disc an undivided oval, unpigmented; frenocular pentagonal, in contact with nasoloreal anteriorly; postoculars three; temporals polygonals, of a medium size; supralabials five; infralabials six; mental wider (2.40 mm) than long (1.10 mm), in contact with first infralabials, postmental posteriorly; postmental single, pentagonal, posterior suture angular, point directed posteriorly, in contact with first and second infralabials; genials in two pairs, anterior pair subquadrangular, in contact with second and third infralabials; posterior genials pentangular, in contact with fourth infralabials laterally; scale rows between genials and collar fold (along midventral line) 17; posteriormost gular row enfolded posteriorly, concealing two granular scale rows; lateral neck scales rounded, conical. Dorsal scales granular laterally and dorsally, scattered conical tubercles on both flanks of body are posteriorly projected, dorsal keeled scales 33 in a longitudinal count, forming four rows from the post occipital region to the insertion of the forelimbs, scattered at the rest of dorsum and becoming four rows again at the insertion of hind limbs, separated by granular scales; transverse dorsal count (enlarged rows at midbody) at fifth transverse ventral scale row 48, at 10th transverse ventral scale row 40, at 15th transverse ventral scale row 38; lateral scales on body near insertion of forelimb small, conical dorsally, mostly granular; ventrals squarish and juxtaposed; complete longitudinal ventral count 23; longitudinal ventral scale rows at midbody 7; 47 scales around midbody; anterior preanal plate scales two; posterior preanal plate scales three; dorsal and dorsolateral surface of tail with at least 62 whorls of enlarged keeled scales; midventral subcaudals squarish, smooth. Limbs pentadactyl; digits clawed; forelimb reaching anteriorly to fifth infralabial; anterolateral and dorsal brachial scales keeled, imbricate; midbrachial anterodorsal scale at least twice as large as adjacent scales, slightly keeled; anteroventral, ventral, and posteroventral scales granular, imbricate, conical; antebrachial scales polygonal, keeled; medial antebrachial scales small, polygonal, smooth; dorsal manus scales polygonal, imbricate, smooth; palmar scales small, polygonal, smooth; dorsal scales on fingers smooth, quadrangular, covering dorsal half of digit, overhanging supradigital scales, two on I, seven on II, ten on III, twelve on IV, 9/8 on V; subdigital scales 6/5 on I, 9/10 on II, 15/14 on III, seventeen on IV, 11/10 on V; dorsal thigh scales granular, some scales bearing conical tubercles, anterodorsal thigh scales polygonal, largest than adjacent scales, slightly keeled; posterodorsal thigh scales small, granular, dorsalmost scales tuberculate, arranged irregularly, ventral thigh scales rounded, smooth, several times smaller than anterodorsal thigh scales; anterior and anteromedial shank scales granular, yuxtaposed, some scales bearing conical tubercles, anteriormost scales at the same size than lateral, posterolateral, and posteromedial shank scales; lateral, posterolateral, and posteromedial shank scales granular, juxtaposed, some bearing conical tubercle; scales on dorsal surface of digits single, quadrangular, smooth, overhanging supradigital scales, four on I, 8/9 on II, thirteen on III, 18/19 on IV, 10/9 on V; subdigital scales single or double, 9/8 on I, 10/11 on II, 18/17 on III, 26/23 on IV, 13/14 on V; femoral pores 20–21.

The completely everted hemipenis is an acapitate organ without a medial welt; apex with two large protrusions separated by the distal end of the sulcus spermaticus; sulcus spermaticus single, flounces lacking calcified spines and forming two chevrons on distal half of hemipenis; sulcate flounces about as wide as asulcate flounces; asulcate flounces becoming shorter distally, five in the basal half and thirteen in each protrusion, distal chevrons separated by a small expansion pleat; sulcus spermaticus single, flanked by a broad naked expansion pleat widened distally.

#### Coloration in preservative.

Dorsal surface of head, dorsal surface of body, tail, limbs, hands and feet dark brown; lateral ocelli present in two pairs with a white rounded center; labial region, throat, chest and venter pale blue with scattered black blotches. Ventral surfaces of forelimbs pale yellow with black blotches; ventral surfaces of thighs pale brown with black blotches above position of femoral pores; ventral surfaces of hands and feet pale brown becoming darker at fingers III, IV and V; ventral surface of tail pinkish brown with diffuse black blotches.

#### Coloration in lif.

(Fig. 1a–d). Dorsal and lateral surfaces, of the head dark brown; rostral and first supralabial scale same color as head; superior labium is bluish with dark spots from second supralabial; iris reddish gold; ventral surface of head, pregular and gular region black with pale blue irregular blotches. Dorsal surface of body same color as head, darker than flanks; lateral surface of body brown with a pair of black ocelli on both sides, before and after insertion of forelimbs, each ocelli bearing a white center, coinciding with a conical tubercle; tuberculate scales darker than granular scales; ventral surface of body same color as ventral surface of head. Limbs, similar to body, ventral surface of arms yellowish brown, ventral surface of legs creamy brown. Coloration of dorsal surface of tail like that of body, ventral surface of tail reddish brown, only red at the base and.

#### Variation.

(Fig. 1, d). In the type series, azygous scales (between frontonasal and prefrontal scales) are present in six specimens (CORBIDI 08324-28, 08335) including males and females, and are absent in five specimens (CORBIDI 06957, 08322, 08334, 08336, 08338); infralabials usually five, four present in CORBIDI 06957, 08324, 08327 and six in CORBIDI 08322, 08325; lateral ocelli are present in two pairs, first pair located anteriorly to the insertion of forelimbs and the second one posteriorly, the white spot at the middle of the ocelli includes usually one conical tubercle; with two conical tubercles at the right side in CORBIDI 08327 and at both sides in CORBIDI 08338. CORBIDI 06957, 08324, 08327, 08334-35 have more than two pairs of lateral ocelli and CORBIDI 08336, 08338 lack of lateral ocelli; in life, ventral coloration in males is usually pale blue, with black blotches in CORBIDI 08322, and yellow with black blotches in CORBIDI 08324, in females the throat and chest can be creamy white or dark brown, belly creamy white or darker bearing or lacking dark blotches. Sexual dimorphism is evident in females, because all of them are lacking femoral pores, furthermore other differences between females and males are the SVL (maximum SVL in females 56 mm, maximum SVL in males 68 mm) and the head width (Maximum head width in females 3.2 mm and maximum head width in males 13.23 mm). See Table 1 for variation in selected morphometric and squamation characters in the specimens examined.

**Table 1.** Morphometric and pholidosis characters in *Potamites montanicola*. Individuals measured include: seven males and three females, all adults. Range is followed by mean value and standard deviation in parenthesis.

		**a (n=10)*Potamites montanicol***
Max SVL (mm)	males	68.6
	females	56.1
Head length/Head width	males	1.4-1.6 (1.53+0.06)
	females	3-4.6 (3.59+0.87)
Number of femoral pores	males	19-21 (20.28+0.75)
	females	0 (0.00+0.00)
Azygous scales		0-1 (0.60+0.51)
Number of genials		3 (3.00+0.00)
Number of postparietals		10-11 (10.30+0.48)
Number of scales around midbody		42-50 (46.10+2.84)
Longitudinal dorsal count		32-37 (33.80+1.81)
Number of longitudinal ventral scale rows		21-23 (21.70+0.67)
Number of transversal ventral scale rows		6-8 (0.90+0.99)
Lamellae under 4th finger		13-17 (16.20+1.31)
Lamellae under 4th toe		22-27 (24.80+1.54)

#### Etymology.

The specific epithet ‘montanicola’ is a compound from the spanish word “montano”, adjective to describe something from a mountain, and the latin suffix “-icola” for “inhabitant” and refers to the montane forests where this species lives.

#### Distribution and natural history.

**Figure 5.**
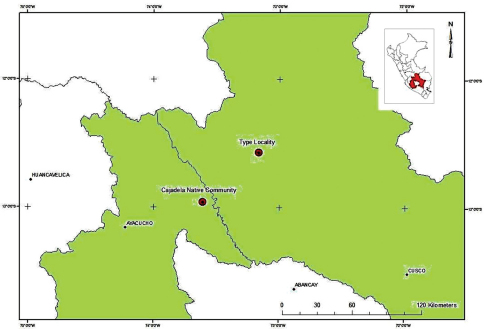
Map showing the type locality and the second site of occurrence (Cajadela Native Community) for *Potamites montanicola*.

*Potamites montanicola* is known from two localities in the Andes in southern Peru (Fig. 5), both separated by 64 km air line and located at the Cordillera de Vilcabamba and Apurimac river valley, the known altitudinal range is between elevations 1570 and 2100 m. The holotype and most of the specimens of the type series were found on the sides of a stream, which were 3 meters wide with stones and rocks as substrate. The vegetation in the area was riverside vegetation mainly composed of: *Miconia* sp., *Gordonia sp.* and *Guarea sp*. and herbs from the family Rubiaceae and Melastomataceae. Climbers (vines and lianas) were diverse and relatively common and include species of the family Celatraceae, Polygalaceae and Campanulaceae. All individuals were found perching on rocks and stones at sides of the stream at night. In some cases, individuals were observed swimming in the middle of the stream, or using the stream to escape. No other lizard species were recorded at the type locality, but on the same stream we observed the vipers *Bothriopsis taeniata* and *Lachesis muta*. Amphibians also reported here include *Hypsiboas balzani*, *Hyalinobatrachium bergeri*, *Osteocephalus mimeticus*, *Pristimantis rhabdolaemus* and *Pristimantis mendax*. The second locality where *Potamites montanicola* was collected (specimen CORBIDI 06957) is a secondary forest, close to the Chiquintirca – Cajadela road. In this site, arboreal vegetation includes species of *Cecropia* sp., and abundant bushes. The specimen CORBIDI 06957 was found during the day near a creek with substrate mainly composed of leaf litter and fallen trunks. In this locality, *Potamites montanicola* is sympatric with the tropidurid lizard *Stenocercus torquatus* and the anurans *Hyalinobatrachium bergeri*, *Hypsiboas balzani*, *Pristimantis mendax* and *Pristimantis rhabdolaemus*. No snakes were reported.

#### Remarks.

The genus *Potamites* is composed of species that are primarily lowland distributed. One of these, *Potamites ecpleopus*, has the largest distribution range in *Potamites* (despite its unclear taxonomy). Sinitsin (1930) assigned the populations of *Potamites* in Perené and Chanchamayo valleys, central Peru, as paratypes of *Potamites ocellatus*, but later, Vanzolini (1995) assigned them as part of the *Potamites ecpleopus* complex and not as *Potamites ocellatus* sensu stricto. *Potamites ocellatus* was then validated and redescribed from only one specimen from El Beni, Bolivia (Vanzolini 1995). These taxonomic uncertainties render the species assignment of the populations from Chanchamayo and Perené unclear. Several surveys to Chanchamayo and Perené from 2008 to 2010 by the senior author resulted in unsuccessful efforts to find the populationsmentioned by Sinitsin (1930). Even though *Potamites montanicola* most northern locality (Cajadela Community) is 250 Km air line from Chanchamayo and Perené valleys, *Potamites montanicola* has a higher vertical distribution range than those populations (by 1000 m). This evidence, along with morphological characteristics, distinguish and validate *Potamites monticola* as distinct. Furthermore, *Potamites montanicola* is the only species described for Peruthat occurs above 2000 meters of elevation and to be reported as exclusive from montane forests. Further studies on the taxonomic identity and the populations of *Potamites ecpleopus* would help to clarify their status and to determine if they belong to a described or undescribed species.

## Supplementary Material

XML Treatment for Potamites montanicola

## Appendix 1

Specimens examined:

*Potamites ecpleopus*.- Peru: AMAZONAs: Condorcanqui, Cordillera de Kampankis, corbidi 09436, 09509, 09516, 09563-67, 09576-78; Cusco: La Convencion, Comunidad Nativa Tangoshiari CORBIDI 0311-13; Pongo de Mainique – Santuario Nacional Megantoni CORBIDI 05519; Campamento Kinteroni CORBIDI 06992-93, 06997; Comunidad Nativa Monte Carmelo CORBIDI 08331.33; Comunidad Nativa Chokoriari CORBIDI 08498-99; lORETO: Datem del Marañon, Andoas CORBIDI 01077, 04637, 04641-43, 04746, 04751, 04981, 05056; Loreto, San Jacinto CORBIDI 01208-09; Singasapa CORBIDI 06529; Rio Corrientes CORBIDI 02731; Río Yanayacu CORBIDI 05989; Maynas, Aguas Negras CORBIDI 0280; Redondococha CORBIDI 0286; Requena, Sierra del Divisor CORBIDI 02246-48, 02585, 04138, 04144, SAN MARTIN: Moyobamba, Comunidad Morro de Calzada CORBIDI 01360; Picota, Chambrillo CORBIDI 08834, Lamas, Pongo de Cainarachi CORBIDI 09059.

*Potamites strangulatus strangulatus*.- PERU: AMAZONAS: Condorcanqui, Cordillera de Kampankis, CORBIDI 09352, 09397, 09399, 09411, 09523-24.

*Potamites strangulatus trachodus*.- Peru: AMAZONAS: Bagua, Chonza Alta CORBIDI 0739-0744; CAJAMARCA: San Ignacio, Alto Ihuamaca CORBIDI 0878-80; SAN MARTIN: Moyobamba, Comunidad Nativa Paitoja CORBIDI 01237-41, 01262-63, 03145, 03147, 03192; Fundo Pabloyacu CORBIDI 01392; Rioja, Zona Reservada Miskiyacu CORBIDI 01429-30, 01434, 01437, 01441, 03274-75; Tarapoto, Cordillera Escalera CORBIDI 06366-69, 06383, 06773.
